# Behavioural Effects of Using Sulfasalazine to Inhibit Glutamate Released by Cancer Cells: A Novel target for Cancer-Induced Depression

**DOI:** 10.1038/srep41382

**Published:** 2017-01-25

**Authors:** Mina G. Nashed, Robert G. Ungard, Kimberly Young, Natalie J. Zacal, Eric P. Seidlitz, Jennifer Fazzari, Benicio N. Frey, Gurmit Singh

**Affiliations:** 1 Department of Pathology & Molecular Medicine, McMaster University, Hamilton, ON, Canada; 2 Michael G. DeGroote Institute for Pain Research and Care, McMaster University, Hamilton, ON, Canada; 3 Department of Psychiatry and Behavioural Neurosciences, McMaster University, Hamilton, ON, L8N 3K7, Canada; 4 Mood Disorders Program and Women’s Health Concerns Clinic, St. Joseph’s Healthcare Hamilton, ON, L8P 3K7, Canada

## Abstract

Despite the lack of robust evidence of effectiveness, current treatment options for cancer-induced depression (CID) are limited to those developed for non-cancer related depression. Here, anhedonia-like and coping behaviours were assessed in female BALB/c mice inoculated with 4T1 mammary carcinoma cells. The behavioural effects of orally administered sulfasalazine (SSZ), a system x_c_^−^ inhibitor, were compared with fluoxetine (FLX). FLX and SSZ prevented the development of anhedonia-like behaviour on the sucrose preference test (SPT) and passive coping behaviour on the forced swim test (FST). The SSZ metabolites 5-aminosalicylic acid (5-ASA) and sulfapyridine (SP) exerted an effect on the SPT but not on the FST. Although 5-ASA is a known anti-inflammatory agent, neither treatment with SSZ nor 5-ASA/SP prevented tumour-induced increases in serum levels of interleukin-1β (IL-1β) and IL-6, which are indicated in depressive disorders. Thus, the observed antidepressant-like effect of SSZ may primarily be attributable to the intact form of the drug, which inhibits system x_c_^−^. This study represents the first attempt at targeting cancer cells as a therapeutic strategy for CID, rather than targeting downstream effects of tumour burden on the central nervous system. In doing so, we have also begun to characterize the molecular pathways of CID.

Depression is commonly reported by cancer patients^1^, and increases mortality in this population^2^. Accordingly, the effective management of depression is essential to improving both quality of life and survivorship in cancer patients. Although late-stage cancer patients are far more likely than the general population to be prescribed an antidepressant^3^, a recent meta-analysis did not find a significant difference in efficacy between antidepressants and placebo in treating cancer patients with depression symptoms^4^. This study, in addition to previous systematic reviews, emphasizes the scarcity of high quality evidence for the effect of antidepressants in cancer-induced depression (CID)^4^,^5^,^6^,^7^.

To investigate the underlying pathophysiology of CID and to explore novel targeted therapies, we have recently developed a validated mouse model of CID^8^. In this model, BALB/c mice that were subcutaneously inoculated with 4T1 mammary carcinoma cells exhibited similar behavioural and neurostructural deficits to those associated with a chronic stress-induced depressive-like state.

Glutamate dysregulation has been strongly linked to depressive disorders. For instance, glutamate is elevated in the plasma of patients with MDD^9^, and magnetic resonance spectroscopy (MRS) studies have revealed a decreased glutamate/glutamine (Glx) and glutamate (Glu) signals in brain regions that are relevant to depression, such as the prefrontal cortex and anterior cingulate cortex^10^,^11^. Moreover, ketamine, a glutamate receptor antagonist, has a rapid antidepressant effect in treatment-resistant patients and preclinical models of depression^12^,^13^,^14^.

Although the underlying biological basis of CID is not yet established, evidence suggests that glutamate signaling may be involved. Glutamate released by the glutamate/cystine antiporter system x_c_^−^ from glioma cells is sufficient to directly induce excitotoxic cell death through chronic glutamate receptor activation of nearby neurons^15^. We have previously reported that multiple breast and prostate cancer cell lines secrete significant amounts of glutamate into the extracellular environment through system x_c_^−^16,17. Although peripheral glutamate does not cross the blood-brain barrier (BBB) under normal conditions^18^, pathological conditions may increase BBB permeability and allow for bidirectional glutamate transport. For example, breast cancer cells have been shown to release high levels of substance P^19^ and cytokines, including IL-1β, IL-6, IL-17A, and TNF-α^20^. Both substance P^21^ and cytokines that are associated with depression and cancer have been shown to disrupt BBB integrity^22^,^23^,^24^,^25^. Intravenous administration of glutamate decelerates the elimination of glutamate in the brain parenchyma^26^. Therefore, irrespective of BBB integrity, excess peripheral glutamate may also cause an accumulation of glutamate in the brain through decreased brain-to-blood glutamate efflux. Therefore, it is plausible that inhibiting glutamate release by peripheral cancer cells would impact CNS glutamate regulation and produce an antidepressant effect.

Recently, it was shown that adult mice deficient in system x_c_^−^ exhibited reduced anxiety- and depressive-like behaviours^27^, which further supports the role of glutamatergic dysregulation in depression, and identifies system x_c_^−^ as a potential therapeutic target. In the present study, we hypothesize that chronic pharmacological inhibition of peripheral cancer cell system x_c_^−^ through oral sulfasalazine (SSZ) treatment will prevent depressive-like behaviours in our CID model. To test our hypothesis, we used fluoxetine (FLX) treatment to establish a positive control group of treated CID. Although only intact SSZ inhibits system x_c_^−^, orally ingested SSZ is predominantly cleaved into 5-aminosalicylic acid (5-ASA) and sulfapyridine (SP) in the gut^28^,^29^. 5-ASA is a known anti-inflammatory drug^30^, and may therefore independently impact depressive behaviours. To clarify the mechanism of any observed antidepressant-like effects of SSZ, a comparison with the effects of its metabolites, 5-ASA and SP, was included. Additionally, we quantified serum levels of IL-1β, IL-6, IL-17A, and TNF-α to provide further insight into the anti-inflammatory impact of chronic treatment with SSZ and its metabolites.

## Materials and Methods

### Cell culture

4T1 murine mammary carcinoma cells (American Type Culture Collection, Manassas, VA, USA) and TM40A murine non-tumourigenic mammary epithelial cells (kindly supplied by Dr. Joseph Jerry, University of Massachusetts, Amherst, MA, USA) were maintained according to supplier specifications. Culturing methods are detailed in “Supplementary Materials and Methods”.

### Cell treatment

The system x_c_^−^ inhibitor SSZ (Sigma-Aldrich) was prepared in accordance with manufacturer’s recommendations in 1 M NH_4_OH to produce a stock solution of 0.1 M SSZ. Control cells were treated with vehicle 1 M NH_4_OH.

### Glutamate release through system x_c_^−^

Glutamate release through system x_c_^−^ was quantified using the cellular uptake of radiolabeled ^14^C-cystine. In cancer cells, cystine uptake is coupled with glutamate release mediated by a 1:1 exchange via system x_c_^−^31. Therefore, measuring the uptake of radiolabeled ^14^C-cystine provides a proportional measure of glutamate release. Furthermore, the specificity of this assay in assessing system x_c_^−^ activity is supported by xCT knockdown results. An approximate 2-fold knockdown of xCT mRNA expression in human breast cancer cells (MDA-MB-231) causes an approximate 2-fold decrease in ^14^C-cystine uptake (unpublished data).

The cystine uptake protocol was adapted from previous reports^32^,^33^. Briefly, 250,000 cells were seeded in 6-well plates. All wells were incubated with ^14^C-cystine, either in the presence or absence of 200 μM of SSZ, for 30 minutes. Cells were then lysed, and the lysate was analyzed using a scintillation counter to quantify radioactivity. The lysate was also used to quantify the amount of protein for each well, and cystine uptake was normalized to total protein. Assay procedures are further detailed in “Supplementary Materials and Methods”.

### Mice

Fifty-nine female BALB/c mice aged 4–6 weeks were obtained from Charles River Laboratories (St. Constant, QC, Canada). Mice were single-housed in cages maintained at 24 °C with a 12-h light/dark cycle, and were provided *ad libitum* access to food and water. Single housing eliminates the protective effect of group housing on depressive-like behaviours observed in tumour-bearing mice^34^. All animal procedures were performed according to guidelines established by the Canadian Council on Animal Care under a protocol reviewed and approved by the *Animal Research Ethics Board* of McMaster University.

### Tumour cell inoculation

Tumour cell inoculations were performed as previously described^8^. Briefly, mice were inoculated with 15,000 4T1 cells in sterile phosphate buffered saline (PBS) subcutaneously above their right flank. Control mice were sham-inoculated with sterile PBS. Mice were weighed weekly (Supplementary Materials and Methods, Figure S1) and tumour size was monitored using a digital caliper every 3–4 days once tumours became palpable at day 8 (Supplementary Materials and Methods, Figure S2). All mice were euthanized 28–29 days after cancer cell inoculation.

### Drug treatments

All drugs were administered *ad libitum* to the mice in place of their normal drinking water. This method of drug treatment was chosen to minimize repeated stressful injections and excessive handling, which might confound the behavioural results. Drug treatments were initiated on the day of tumour cell inoculation and continued until endpoint.

FLX hydrochloride (Sigma-Aldrich) was dissolved in drinking water at a concentration of 100 μg/mL to obtain a target mean drug dose of approximate 20 mg/kg/day, which is the highest chronic dose reported to be effective in mice^35^,^36^. SSZ was dissolved in a small volume of 1 M NH_4_OH, which was then adjusted to pH 8.5 using 1 M HCl. The concentrated SSZ solution was then diluted 1:30 in drinking water to a final concentration of 0.3 mg/mL. In humans, only approximately 12% of orally ingested SSZ escapes colonic cleavage and is absorbed into systemic circulation^29^. Based on these bioavailability data, mean water consumption, and mean weight of mice, it was estimated that 0.3 mg/mL of SSZ would translate into a dose of approximately 8 mg/kg/day. This target dose was chosen to be comparable to a dose that we have previously demonstrated to effectively reduce cancer-induced bone pain^37^. The SSZ metabolites, 5-ASA and SP (Sigma-Aldrich), were prepared the same way as SSZ to a final concentration of 0.09 mg/mL for 5-ASA and 0.15 mg/mL for SP. These target concentrations were chosen to match the expected concentrations of metabolites produced from oral treatment with 0.3 mg/mL of SSZ. As such, a liberal estimation of 80% colonic cleavage of SSZ into equal parts 5-ASA and SP was assumed^28^ and concentrations were adjusted based on the molecular weights of each metabolite.

FLX and SSZ are known to have an aversive taste. Therefore, all vehicles included 2% orange extract (McCormick Canada Inc., London, ON, Canada) to increase the palatability of drug solutions. Sweetening the water was avoided in order to maintain sucrose preference test (SPT) sensitivity at post-treatment measurement. All drugs were administered in opaque bottles for light protection.

### Behavioural analyses

Behavioural tests were performed as previously described^8^. Anhedonia was evaluated using the sucrose preference test (SPT) and active versus passive coping behaviour was evaluated using the forced swim test (FST) and the tail suspension test (TST). For the SPT, mice were first habituated to sucrose solution and a 2-bottle setup in their home cages for 72 h. For baseline and post-treatment testing, mice were presented with a two-bottle option of water and 3% sucrose solution for 48 h. At 24 h, the positions of the two bottles were switched to reduce location bias. Bottles were weighed before and after testing, and preference was calculated as the percentage of sucrose solution intake relative to total fluid intake^38^.

The FST and TST were performed using automated systems (BioSeb, Vitrolles, France). For the FST, mice were individually placed into beakers of water. For the TST, mice were suspended by their tails, and strain gauge input was used to assess mobility. For both tests, the total duration was 6 minutes, with the first minute of the test discounted from analysis to allow for behavioural stabilization^39^,^40^. Immobility time was the primary outcome measure, and power of movement (PM) was reported as a secondary measure for the TST. Although this study utilizes female mice, it has been demonstrated that estrous cycle does not impact sucrose preference or coping behaviours in healthy or tumour-bearing female mice^34^.

### Experimental groups

Five groups of mice were randomly assigned to treatments: healthy + vehicle control (n = 11), tumour + vehicle control (n = 12), tumour + FLX (n = 12), tumour + SSZ (n = 12), and tumour + combination 5-ASA/SP (n = 12).

### Euthanasia and tissue harvesting

Euthanasia was performed by isoflurane anesthesia and cardiac puncture exsanguination for endpoint blood collection, followed by decapitation. Blood samples were collected intracardially using sterile 25 G needles and 1 mL syringes. Blood samples were allowed to coagulate for 30–60 minutes at room temperature, and then centrifuged to extract serum^41^. Brains were immediately removed, rinsed and placed in sterile Hanks’ Balanced Salt Solution (HBSS) for further processing (see *Brain Metastases* below).

### Brain Metastases

Brain metastases were investigated using a clonogenic assay as outlined by Pulaski and Ostrand-Rosenberg (2001). This assay exploits the fact that, unlike mouse host cells, 4T1 tumour cells are resistant to 6-thioguanine (6-TG). Briefly, brains were minced and dissociated in a cocktail of collagenase type IV and elastase in HBSS. Samples were cultured in complete media containing 60 μM 6-TG for 10–14 days. Cells were then fixed with methanol and stained with methylene blue, and the number of colonies were quantified. Clonogenic assay procedures are further detailed in “Supplementary Materials and Methods”.

### Serum Glutamate

Serum proteins were precipitated from samples using 1 volume of chilled 100% (w/v) trichloroacetic acid (TCA) to 3 volumes of serum. Samples were centrifuged and the supernatant removed and neutralized with potassium hydroxide (KOH). Protein pellets were quantified for each sample using the BioRad assay as described in “Supplementary Materials and Methods”. Serum glutamate in the supernatant was analyzed using the AMPLEX Red glutamic acid assay kit (Invitrogen/Molecular Probes, Eugene, OR, USA) and analyzed on a CytoFluor Series 4000 Fluorescence Multi-Well Plate Reader (PerSeptive Biosystems, Framingham, MA, USA). Serum glutamate was normalized to total serum protein for each sample.

### Serum Cytokines

Serum samples from each mouse were sent to Eve Technologies (Calgary, AB, Canada) for cytokine quantification of IL-1β, IL-6, IL-17A, and TNF-α. Cytokine quantification is further detailed in “Supplementary Materials and Methods”.

### Data Analyses

Three independent planned pairwise comparisons were used to analyze results from the ^14^C-cystine uptake assay: 4T1 vs. TM40A, TM40A vs. TM40A + 200 μM SSZ, and 4T1 vs. 4T1 + 200 μM SSZ. For each comparison, results from 3 independent experiments (each performed in triplicates) are represented as mean fold-changes in counts per minute (CMP)/mg protein relative to controls. All pairwise comparisons were evaluated using one-tailed Student *t*-tests.

One-way analysis of variance (ANOVA) was used to analyze the behavioural data (SPT, FST, and TST), serum glutamate, and serum cytokines. For these data, 4 planned comparisons were analyzed. Control mice were compared with tumour mice to establish benchmark tumour-induced changes for each assay. Tumour mice were compared with each intervention group (FLX, SSZ, and 5-ASA) to explore drug effects on each assay. The Holm-Šidák correction was used to correct for multiple comparisons.

In this investigation we used a peripheral tumour model for CID. Therefore, we are primarily interested in attenuating depressive behaviours by targeting peripheral tumour cells. However, 4T1 cells are capable of metastasizing to the brain^42^. Therefore, as a secondary measure for all behavioural data, linear regression analyses were performed to investigate whether brain metastases were predictive of tumour-induced or drug-induced behavioural changes.

For all analyses, the significance level was set at α = 0.05. Grubb’s test was used to identify and exclude any outliers. The Shapiro-Wilk test of was used to determine normality. Data that was not normally distributed was subsequently transformed to achieve approximate normality prior to further statistical considerations. Data are presented as mean ± the SEM. Statistical analyses were performed using GraphPad Prism software version 6.0 for Macintosh (GraphPad Software, Inc., La Jolla, CA, USA). All graphs were prepared on Microsoft Excel 2011 version 14.1.0 (Microsoft, Redmond, WA, USA).

## Results

### Glutamate release through system x_c_^−^

**Figure 1:**
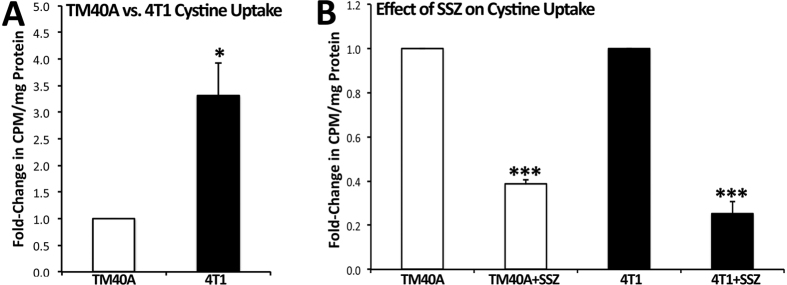
^14^C-cystine uptake assay on 4T1 cancer cells and TM40A mammary epithelial cells, both derived from BALB/c mice. (**A**) Cystine uptake by 4T1 cells was more than 3-fold higher than TM40A cells (*P* = 0.032). (**B**) 200 μM SSZ caused a decrease in TM40A cystine uptake to 0.39-fold (*P* < 0.001) and a decrease in 4T1 cystine uptake to 0.25-fold (*P* < 0.001). Results are represented as means from 3 experiments (performed in duplicates) ± SEM. Statistical significance was assessed through one-tailed Student *t*-tests. *P < 0.05. ***P < 0.001.

Cystine uptake, and consequently glutamate release, by tumourigenic 4T1 cells was more than 3-fold higher than that observed by non-tumourigenic TM40A control cells (*P* = 0.032; Fig. 1A). Treatment with 200 μM of SSZ decreased cystine uptake to 0.39-fold for TM40A cells (*P* < 0.001) and decreased cystine uptake to 0.25-fold for 4T1 cells (0.25-fold) (*P* < 0.001; Fig. 1B).

### Behavioural analyses

Mice in the tum+FLX group consumed a mean FLX dose of 21.59 ± 0.56 mg/kg/day. Mice in the tum+SSZ group consumed a mean SSZ dose of 71.41 ± 2.98 mg/kg/day, or 8.57 ± 0.36 mg/kg/day when corrected for systemic absorption of intact SSZ^29^. Mice in the tum+5-ASA/SP group consumed a mean 5-ASA dose of 20.32 ± 0.54 mg/kg/day, and a mean SP dose of 33.86 ± 0.89 mg/kg/day.

**Figure 2:**
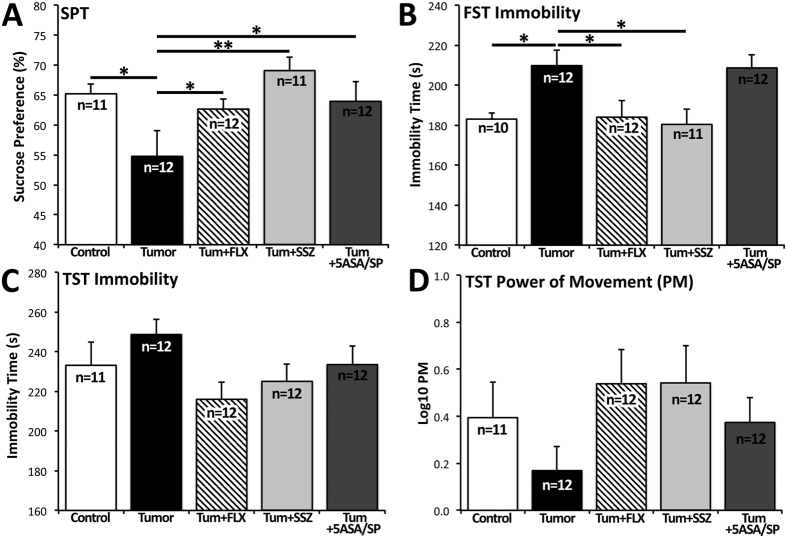
Behavioural results for the 5 experimental groups: control, tumour, tum+FLX, tum+SSZ, tum+5-ASA/SP. (**A**) In the sucrose preference test (SPT) preference is calculated as (sucrose water consumption/total fluid intake) × 100, where 50% represents equal consumption of sucrose water and regular water. Tumour mice showed a lower sucrose preference compared to control (*P* = 0.026). Treatments with FLX, SSZ, and 5-ASA/SP were associated with higher sucrose preference compared to untreated tumour mice (*P* = 0.040, *P* = 0.002, *P* = 0.035, respectively). (**B**) In the forced swim test (FST), tumour mice had higher immobility time compared to control mice (*P* = 0.033). FLX and SSZ were associated with lower immobility times compared to untreated tumour mice (*P* = 0.033, *P* = 0.019, respectively). (**C**) In the tail suspension test (TST), no significant changes were observed. Trends indicated a lower immobility time and higher log_10_ PM associated with FLX and SSZ treatments compared to untreated mice. Data are presented as mean ± SEM. Results were analyzed using one-way ANOVA. The Holm-Šidák correction was used to correct for multiple comparisons. *P < 0.05, **P < 0.01.

In the SPT, lower preference for sucrose solution indicates anhedonia-like behaviour, which is a core symptom of depression characterized by the loss of ability to experience pleasure. Our results revealed a significant main effect of treatment group (F_(4,53)_ = 3.84, *P* = 0.008). As expected, untreated tumour mice showed lower sucrose preference compared to control mice (*P* = 0.026) (Fig. 2A). Treatment with FLX, SSZ, and 5-ASA/SP were all associated with higher sucrose preference compared to untreated tumour mice (*P* = 0.040, *P* = 0.002, *P* = 0.035, respectively).

In the FST and TST, longer immobility times indicate passive coping behaviour, a depressive feature that is sensitive to antidepressant treatment. Our results revealed a significant main effect of treatment group on FST immobility (F_(4,52)_ = 4.36, *P* = 0.004). As expected, untreated tumour mice had higher immobility time compared to control mice (*P* = 0.033) (Fig. 2B). FLX and SSZ were associated with lower immobility times compared to untreated tumour mice (*P* = 0.033, *P* = 0.019, respectively). 5-ASA/SP treatment did not prevent the tumour-induced increase in immobility time.

Two measures for the TST are reported: immobility time (Fig. 2C) and PM (Fig. 2D). PM did not follow a normal distribution. Log_10_ transformation of these data achieved approximate normality. There was no main effect of treatment group on TST immobility (F_(4,54)_ = 1.70, *P* = 0.163) or log_10_ PM (F_(4,54)_ = 1.32, *P* = 0.276). Here we independently replicated our previous finding that the TST is not sensitive in detecting passive coping behaviour in the CID model^8^.

**Figure 3:**
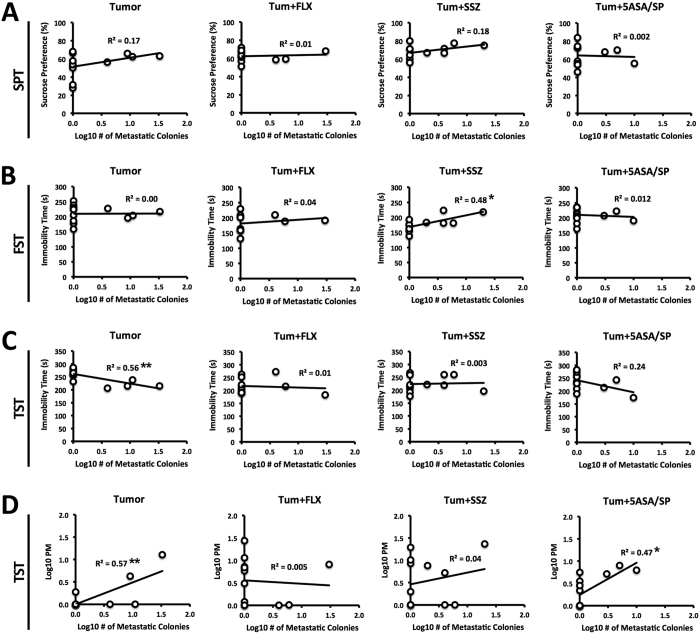
Brain metastasis regression analyses for the 4 tumour groups: tumour, tum+FLX, tum+SSZ, tum+5-ASA/SP. (**A**) Brain metastases were not predictive of sucrose preference for any of the tumour groups. (**B**) Brain metastases were predictive of lower FST immobility time for mice treated with SSZ (*P* = 0.019). (**C**) Brain metastases were predictive of lower immobility time for untreated tumour-bearing mice (*P* = 0.005). (**D**) Brain metastases were predictive of higher PM for untreated tumour mice (*P* = 0.004) and 5-ASA/SP treated mice (*P* = 0.013). Results were analyzed using linear regression analyses. *P < 0.05, **P < 0.001.

Approximately 35% of tumour-bearing mice developed brain metastases 4 weeks after inoculation, which is consistent with previous observations^42^. Among mice that developed brain metastases, the number of metastatic colonies was not normally distributed. Log_10_ transformation of this data achieved approximate normality. Subsequently, regression analyses revealed that brain metastases did not predict sucrose preference in any direction for any of the tumour groups (Fig. 3A). Brain metastases were moderately predictive of increased immobility time on the FST for the tumour group treated with SSZ (*P* = 0.019) (Fig. 3B). A possible explanation for this is that mice with brain metastases were less responsive to SSZ treatment on this measure. In the TST, metastases were moderately predictive of lower immobility time and higher PM for untreated tumour mice (*P* = 0.005 and *P* = 0.004, respectively) (Fig. 3C). This result was counterintuitive, but may help explain the consistent lack of sensitivity of this test in detecting tumour-induced passive coping behaviour and drug effects. In addition, brain metastases were moderately predictive of higher PM for the mice treated with 5-ASA/SP (*P* = 0.013), but did not predict PM for mice treated with SSZ or FLX.

### Serum Glutamate

**Figure 4:**
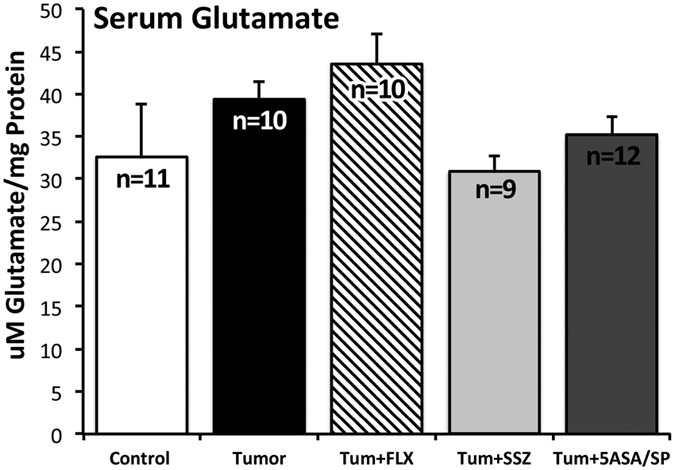
Serum level of glutamate for the 5 experimental groups: control, tumour, tum+FLX, tum+SSZ, tum+5-ASA/SP. Data are presented as mean ± SEM. No significant differences were observed between treatment groups. Trends were consistent with the expected increase of serum glutamate in tumour-bearing mice, and the expected inhibition of cancer cell glutamate release by SSZ. Results were analyzed using one-way ANOVA. The Holm-Šidák correction was used to correct for multiple comparisons.

There was no main effect of treatment group on serum glutamate (F_(4,47)_ = 1.87, *P* = 0.132). However, trends observed in serum glutamate levels are consistent with the expected glutamate release by 4T1 cells in tumour-bearing mice, and with the hypothesized inhibition of this glutamate release by SSZ (Fig. 4).

### Serum Cytokines

**Figure 5:**
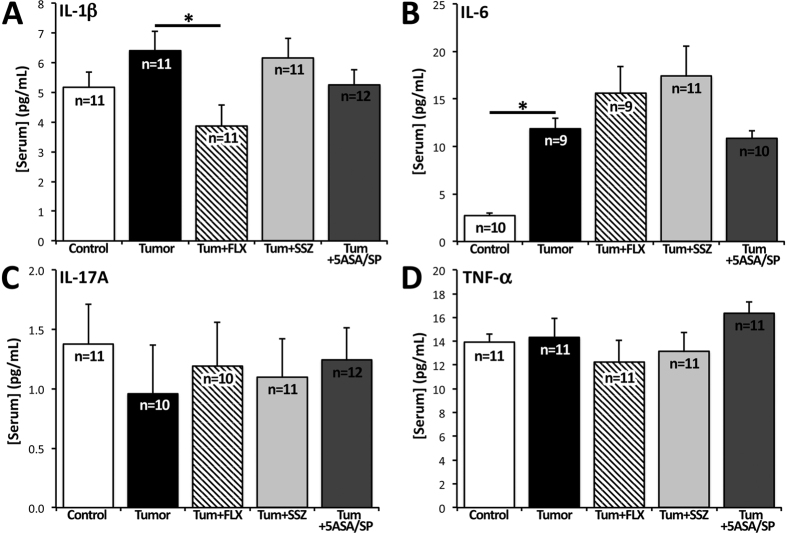
Serum level of pro-inflammatory cytokines for the 5 experimental groups: control, tumour, tum+FLX, tum+SSZ, tum+5-ASA/SP. (**A**) FLX-treated mice had a significantly lower level of serum IL-1β compared to untreated tumour mice (*P* = 0.022). (**B**) Untreated tumour-bearing mice had higher levels of serum IL-6 compared to control mice (*P* = 0.015). (**C**) Serum IL-17A did not differ between any experimental groups. (**D**) Serum TNF-α did not differ between any experimental groups. Data are presented as mean ± SEM. Results were analyzed using one-way ANOVA. The Holm-Šidák correction was used to correct for multiple comparisons. *P < 0.05.

There was a significant main effect of treatment group on IL-1β serum level (F_(4,51)_ = 2.62, *P* = 0.046). FLX-treated mice had a significantly lower level of serum IL-1β compared to untreated tumour mice (*P* = 0.022) (Fig. 5A). Serum levels of IL-1β did not differ for mice treated with SSZ or 5-ASA/SP compared to untreated tumour mice. There was a significant main effect of treatment group on IL-6 serum level (F_(4,44)_ = 7.79, *P* < 0.0001). Untreated tumour mice had higher levels of serum IL-6 compared to control mice (*P* = 0.015) (Fig. 5B). Serum levels of IL-6 did not differ for mice treated with FLX, SSZ or 5-ASA/SP compared to untreated tumour mice. IL-17A and TNF-α levels were not different between groups (Fig. 5C and D).

## Discussion

In the present study, murine 4T1 mammary carcinoma cells had greater cystine uptake, and consequently greater glutamate release, compared to non-tumourigenic TM40A mammary epithelial cells derived from the same mouse strain (Fig. 1A). Furthermore, we demonstrated that SSZ inhibits cystine uptake in both TM40A cells and 4T1 cells (Fig. 1B), suggesting that system x_c_^−^ is primarily responsible for glutamate release. These results are consistent with previous studies from our group showing that human breast and prostate cancer cell lines release large amounts of glutamate into their microenvironment through the glutamate/cystine antiporter system x_c_^−^16,17. We also found that the use of SSZ (a system x_c_^−^ inhibitor) and FLX (used as a positive control) in tumour-bearing mice exerted antidepressant-like effects, namely increased sucrose preference and decreased FST immobility. Results from all behavioural tests, serum glutamate, and serum cytokine analyses are summarized in Table 1.

To establish a benchmark for antidepressant-like efficacy in the present study, we used the selective serotonin reuptake inhibitor (SSRI) FLX. FLX effectively attenuates depressive-like behaviours in animal models when administered chronically^35^,^36^. SSRIs are useful for treating acute depressive episodes and preventing subsequent episodes in MDD subjects^43^. The rapid progression of tumour growth in our CID model was more suitable for testing the prophylactic efficacy of FLX, SSZ, and 5-ASA/SP, rather than reversal of depressive-like behaviours. In our CID model, chronic treatment with SSZ was at least as effective as FLX in preventing depressive-like behaviours on both the SPT and FST. Treatment with the SSZ metabolites, 5-ASA/SP, prevented the development of anhedonia-like behaviour on the SPT, but did not prevent passive coping on the FST. Since the FST is widely considered to be the gold standard *in vivo* assay for antidepressant detection, this suggests that the observed antidepressant-like effect of SSZ was likely primarily due to intact SSZ, and therefore system x_c_^−^ inhibition. However, given the mixed behavioural results observed for 5-ASA/SP, further investigation is warranted to clarify the differential antidepressant effects of SSZ and its metabolites.

Serum cytokine analysis suggested that neither 70 mg/kg/day of SSZ nor 20 mg/kg/day of 5-ASA were effective at preventing the increase in IL-6 that was induced by tumour burden (Fig. 5A and B). A previous study found that 5-ASA, which is used to treat inflammatory bowel disease, decreased intestinal inflammation at doses as low as 8.3 mg/kg/day in rats^44^. The lack of effect we observed on serum cytokines may be due to the fact that only about 10% of 5-ASA is absorbed systemically, where it is rendered largely inactive by extensive acetylation^45^. Although anti-inflammatory agents, such as celecoxib, can exert antidepressant effects in a subset of individuals with MDD^46^, 5-ASA produced mixed behavioural results in our CID model, perhaps due to its limited absorption. Furthermore, it is worth noting that the present study focused on inflammatory markers that have previously been associated with depression. In light of the behavioural effect of 5-ASA/SP treatment on the SPT despite failure to prevent tumour-associated increases in IL-1β and IL-6, an investigation of a wider array of inflammatory markers might help clarify the observed behavioural effects of these drugs in our model. By contrast, FLX administration was associated with lower serum IL-1β compared to untreated tumour mice (Fig. 5A). This result is consistent with meta-analysis results in MDD showing that FLX reduces serum levels of IL-1β but not TNF-α, with mixed results for IL-6^47^.

Results from the serum cytokine analysis also provide plausibility for a direct depressive mechanism of peripherally released glutamate on the CNS. In our CID model, tumour burden was associated with increased IL-6, and possibly IL-1β. These cytokines have been demonstrated to reduce BBB integrity, thereby increasing permeability^24^,^25^. Future analyses of BBB integrity and systemic administration of radiolabeled glutamate would be useful in better characterizing the impact of peripherally released glutamate on the CNS.

Intraperitoneal (i.p.) administration of SSZ inhibits tumour growth in a glioma model^48^, and affects CNS extracellular glutamate levels in healthy rats^49^. These data suggest that SSZ is able to cross the BBB. Therefore, we cannot rule out the possibility that the observed antidepressant-like behavioural effects of SSZ in the present study are partially attributable to a direct central mechanism. However, it is interesting to note that Lutgen *et al*. did not observe an effect on the FST with up to 16 mg/kg of i.p. SSZ in healthy rats^49^. Therefore, for the present study, it is likely that the observed antidepressant-like effect of SSZ is primarily attributable to peripheral inhibition of tumour-derived glutamate. This is consistent with our observation that intact SSZ, but not 5-ASA/SP, prevented the development of passive coping behaviour on the FST. Furthermore, it has been demonstrated that the anti-tumour effects of SSZ are entirely attributable to system x_c_^−^ inhibition, rather than its reported anti-inflammatory and apoptotic influence through nuclear factor-κB (NF-κB) inhibition^50^.

A limitation in our study is that the serum glutamate analysis did not yield significant results, which may in part be attributable to regulatory mechanisms, or due to the technique we used. Glutamate scavenging by glutamate-oxaloacetate transaminase (GOT) and glutamate-pyruvate transaminase (GPT) would make it difficult to detect dynamic glutamate dysregulation at a single time point^51^. Nevertheless, trends in serum glutamate were consistent with SSZ inhibition of glutamate release by cancer cells (Fig. 4). Although not statistically significant, the slight increase in serum glutamate in tumour mice, and decrease in SSZ treated mice, may be biologically relevant considering the tight regulation of this molecule, both peripherally and centrally. Further investigation into serum glutamate would benefit from the inclusion of more blood collection time points, as well as a more direct method of glutamate measurement, such as high performance liquid chromatography (HPLC). Caution should also be taken when interpreting the results of this study due to the lack of specificity of SSZ to system x_c_^−^. Oral SSZ was chosen for the current investigation due to the clinical relevance of this model. SSZ is a clinically useful and well-tolerated system x_c_^−^ inhibitor^52^. Valuable insight may be derived from further investigation of more specific system x_c_^−^ inhibitors, such as S-4-carboxy-phenylglycine (S-4-CPG)^53^. However, the pharmacokinetics of orally administered S-4-CPG has not been described, and this model would likely require daily intraperitoneal injections or surgically implanted osmotic pumps. Stress induced by either of these modes of drug delivery would need to be carefully considered. Genetically modulating system x_c_^−^ activity represents a more specific method of investigating the impact of cancer cell-derived glutamate on CID. To this end, we are currently developing an xCT knockdown 4T1 cancer cell line to be used in future *in vivo* studies. This cell line will be crucial in determining whether glutamate secretion by peripheral cancer cells is necessarily and/or sufficient to mediate a depressive-like phenotype.

In summary, we have demonstrated that chronic oral administration of SSZ is at least as effective as FLX at preventing the development of depressive-like behaviours in a preclinical model of CID. This finding carries clinical significance, particularly considering recent evidence that SSZ inhibits tumour progression^48^,^50^ and cancer-induced bone pain^37^. Our results are consistent with the hypothesis that the primary mechanism of antidepressant-like action of SSZ may be through inhibition of glutamate release via system x_c_^−^ at the peripheral tumour site. However, this hypothesis warrants further investigation, as previously discussed. FLX was also efficacious in preventing the development of depressive-like behaviours in tumour-bearing mice. It may, therefore, be interesting to investigate the effects of standard antidepressants augmented with SSZ in the treatment of CID. While this investigation focuses on peripheral modulation of glutamate signaling in CID, further assessment of central glutamate signaling, using ketamine and other recently described glutamatergic modulators, would further enhance our understanding of CID. Overall, our results represent a potential new pathway for drug development in the treatment of CID. This approach can target the effects of cancer cells directly to prevent depressive symptoms, rather than targeting downstream effects on the CNS. These results have implications not only for the pathophysiology of CID, but also as a novel preclinical model for the development of more targeted therapy.

## Additional Information

**How to cite this article**: Nashed, M. G. *et al*. Behavioural Effects of Using Sulfasalazine to Inhibit Glutamate Released by Cancer Cells: A Novel target for Cancer-Induced Depression. *Sci. Rep.* **7**, 41382; doi: 10.1038/srep41382 (2017).

**Publisher's note:** Springer Nature remains neutral with regard to jurisdictional claims in published maps and institutional affiliations.

## Supplementary Material

Supplementary Material

## Figures and Tables

**Table 1:** Summary of results for behavioural and cytokine analyses.

Comparison	SPT	FST	TST (Immob.)	TST (P.M.)	Serum Glu	IL-1β	IL-6	IL-17A	TNF-α
Control vs. Tumour	↓	↑	—	—	—	—	↑	—	—
Tumour vs. Tum+FLX	↑	↓	—	—	—	↓	—	—	—
Tumour vs. Tum+SSZ	↑↑	↓	—	—	—	—	—	—	—
Tumour vs. Tum+5-ASA/SP	↑	—	—	—	—	—	—	—	—

“−”Denotes no differences between groups. One arrow denote a mean difference between groups with 0.05 > *P* > 0.01. Two arrows denote a mean difference between groups with *P* < 0.01. The direction of the arrows represents the direction of change of mean for that particular measure. SPT, sucrose preference test; TST, tail suspension test; FST, forced swim test; FLX, fluoxetine; SSZ, sulfasalazine; 5-ASA, 5-aminosalicylic acid; SP, sulfapyridine; Glu, glutamate; IL, interleukin; TNF-α, tumour necrosis factor-α.
