# Supplementary material for: Behavioural Effects of Using Sulfasalazine to Inhibit Glutamate Released by Cancer Cells: A Novel target for Cancer-Induced Depression
Source: Sci Rep. 2017 Jan 25;7:41382. doi: 10.1038/srep41382 (PMC5264609; doi:10.1038/srep41382)
Supplement: Supplementary Material [file srep41382-s1.doc]

**Behavioural Effects of Using Sulfasalazine to Inhibit Glutamate Released by Cancer Cells: A Novel target for Cancer-Induced Depression**

^1,2^Mina G. Nashed, M.Sc., Ph.D.; ^1,2^Robert G. Ungard, M.Sc.; ^1,2^Kimberly Young, B.H.Sc.; ^1,2^Natalie J. Zacal, M.Sc.; ^1,2^Eric P. Seidlitz, M.Sc., Ph.D.; ^1,2^Jennifer Fazzari, B.Sc.; ^3,4^Benicio N. Frey, M.D., M.Sc., Ph.D.; ^1,2^Gurmit Singh, Ph.D.*

*^1^Department of Pathology & Molecular Medicine, McMaster University, Hamilton, ON, Canada*

*^2^Michael G. DeGroote Institute for Pain Research and Care, McMaster University, Hamilton, ON, Canada.*

*^3^Department of Psychiatry and Behavioural Neurosciences, McMaster University, Hamilton, ON, L8N 3K7, Canada.*

*^4^Mood Disorders Program and Women’s Health Concerns Clinic, St. Joseph's Healthcare Hamilton, ON, L8P 3K7, Canada.*

*** Corresponding author.**

**Address:** Department of Pathology & Molecular Medicine, McMaster University, 1280 Main Street West, Hamilton, ON L8N 3Z5, Canada.

**Tel.:** +1-905-525-9140 x 28144

**E-mail address:** singhg@mcmaster.ca

**URL:** http://www.singhlab.ca

Supplemental Materials and Methods

*Cell Culture*

4T1 cells were maintained in Roswell Park Memorial Institute medium (RPMI-1640; Life Technologies, Carlsbad, CA) supplemented with 10% fetal bovine serum (FBS) and 1% antibiotic/antimycotic (Life Technologies). TM40A cells were maintained in Dulbecco's Modified Eagle Medium: Nutrient Mixture F-12 (DMEM:F12; Life Technologies) supplemented with 10% FBS, 10 μg/mL insulin, 1% antibiotic/antimycotic (Life Technologies), and 20 ng/mL mouse Epidermal Growth Factor (mEGF; Sigma-Aldrich, St. Louis, MO, USA). All cells were incubated at 37 °C and 5% CO_2_.

*Glutamate release through system x_c_^-^*

For both 4T1 and TM40A cells, 250,000 cells per well were seeded in a 6-well plate and allowed to adhere overnight. Media was then replaced with 300 μL 1× Hank’s Balanced Salt Solution (HBSS; Invitrogen, Burlington, ON, Canada) containing either vehicle 1 M NH_4_OH or 200 μM of SSZ and 0.45 μL of 20 μCi/mL ^14^C-cystine (PerkinElmer, Waltham, MA, USA) added to each well. Following incubation at 37 °C for 30 minutes, HBSS was aspirated and cells were lysed using lysis buffer (0.1 N NaOH containing 0.1% Triton X-100; Sigma). 100 μL of the lysate was added to 1 mL of Ecoscint-H solution (National Diagnostics, Atlanta, GA, USA) in a scintillation vial. A Beckman LS6500 scintillation counter (Beckman Coulter, Inc, Brea, CA USA) was used to quantify the radioactivity of each vial.

Cystine uptake was normalized to total protein using the BioRad protein assay (BioRad Laboratories, Inc, Hercules, CA, USA). This assay was performed in triplicate in a 96-well plate. A 1:4 dilution of BioRad solution was added to 10 μL samples of lysate and incubated for 5 minutes. The plate was then analyzed using a BioTek PowerWave XL plate reader (BioTek Instruments, Inc, Winooski, VT, USA) at 570 nm. Quantification of protein in each sample allowed for the standardization of the counts per minute (CPM) reading by the scintillator to total protein.

*Mice Weights and Tumour Volume*

All mice were weighed once per week. Weight was expressed as mean body weight for each experimental group, corrected for tumour mass in the tumour group (**Figure S1**). Tumour growth was measured every 3-4 days when tumours became palpable. Growth was expressed as mean tumour mass (in grams) over time (**Figure S2**). Calipers were used to measure tumour length, width, and depth, and hemi-ellipsoid tumour volume was calculated as *V= L* × *W* × *H* × 0.5236 ^1,2^. To correct for tumour mass when measuring body weight, tumour volume was converted to mass, assuming soft-tissue density of 1 g/cm^3 3,4^. For these data, two-way repeated-measures ANOVA (group × experimental days) were used to analyze between-group effects, followed by Tukey’s *post hoc* test for multiple comparisons.

*Brain Metastases*

Brain samples were first rinsed in HBSS to remove surface blood, and then minced using surgical scissors. The minced brains were dissociated in a filter-sterilized cocktail of 2 mg/mL collagenase type IV (Worthington Biochemical, Lakewood, NJ, USA) and 30 units of elastase (MP Biomedical, Santa Ana, CA, USA) in HBSS. Samples were incubated while mixing for 120 minutes at 37 °C, and then filtered through 70-μm nylon cell strainers (Life Technologies) to remove any undigested tissue. Filtered samples were centrifuged and the supernatant discarded, and the resultant pellets were washed and centrifuged in HBSS 2 times. Following the final wash, pellets were resuspended in Iscove's Modified Dulbecco's Media (IMDM) supplemented with 10% FBS, 1% antibiotic/antimycotic (Life Technologies), and 60 μM 6-TG (Sigma-Aldrich) and plated onto 10-cm tissue culture dishes. Dishes were incubated at 37 °C and 5% CO_2_ for 10-14 days. Cells were then fixed in methanol and stained with 0.03% (w/v) methylene blue solution. Colonies were quantified for each dish, with each colony representing one clonogenic metastatic cell.

*Serum Cytokines*

Serum samples were diluted 2-fold in sterile PBS and 75 μL of serum samples were sent to Eve Technologies (Calgary, AB, Canada) for cytokine quantification of IL-1β, IL-6, IL-17A, and TNF-α. Eve Technologies uses Multiplexing LASER Bead Technology with each metabolite corresponding to a unique fluorophore signature. Capture antibodies are coupled with the metabolite’s particular beads and a bead analyzer (Bio-Plex 200) is used to quantify the metabolite.

Figures


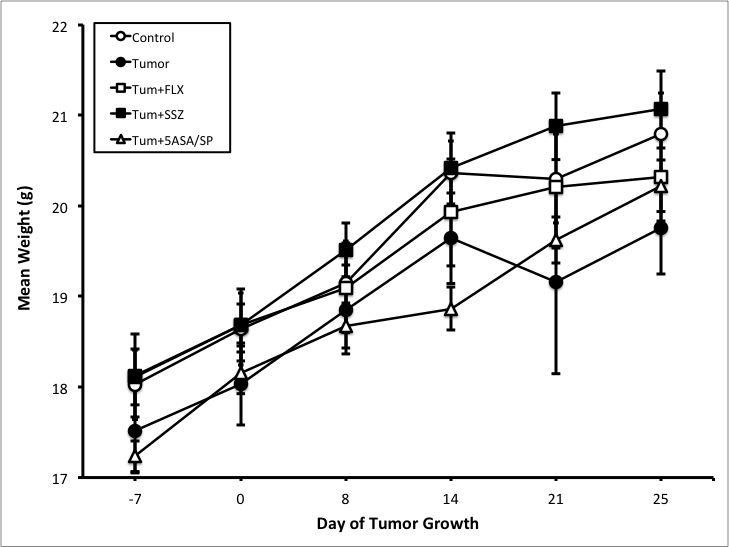


**Figure S1.** Mean body weight of mice in the 5 experimental groups (Control, n = 11; Tumour, n = 12; Tum+FLX, n = 12; Tum+SSZ; n = 12, Tum+5-ASA/SP, n = 12). Data are presented for the duration of tumour growth and weights were corrected for tumour mass. Data are expressed as mean ± SEM. Two-way repeated-measures ANOVA with Tukey’s *post hoc* test for multiple comparisons were used to analyze group differences over time. None of the groups at any time point revealed significant differences in (corrected) weight.


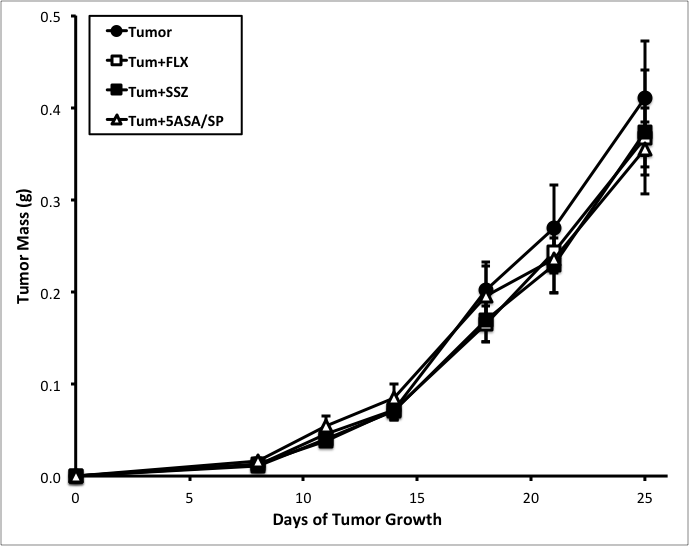


**Figure S2**. Tumour growth in mice subcutaneously inoculated with 15,000 4T1 mammary carcinoma cells (Tumour, n = 12; Tum+FLX, n = 12; Tum+SSZ, n = 12, Tum+5-ASA/SP, n = 12). Tumour growth pattern was exponential and tumour mass reached a cumulative mean of 0.38 ± 0.02 g by day 26 of tumour growth. Data are expressed as mean ± SEM. Two-way repeated-measures ANOVA with Tukey’s post hoc test for multiple comparisons were used to analyze group differences over time. None of the groups at any time point revealed significant differences in tumour size.

References

1 Lein, M. *et al.* Synthetic inhibitor of matrix metalloproteinases (batimastat) reduces prostate cancer growth in an orthotopic rat model. *The Prostate* **43**, 77-82 (2000).

2 Tomayko, M. M. & Reynolds, C. P. Determination of subcutaneous tumor size in athymic (nude) mice. *Cancer chemotherapy and pharmacology* **24**, 148-154 (1989).

3 Jensen, M. M., Jorgensen, J. T., Binderup, T. & Kjaer, A. Tumor volume in subcutaneous mouse xenografts measured by microCT is more accurate and reproducible than determined by 18F-FDG-microPET or external caliper. *BMC medical imaging* **8**, 16, doi:10.1186/1471-2342-8-16 (2008).

4 Montelius, M., Ljungberg, M., Horn, M. & Forssell-Aronsson, E. Tumour size measurement in a mouse model using high resolution MRI. *BMC medical imaging* **12**, 12, doi:10.1186/1471-2342-12-12 (2012).
